# Adaptive Filtering Methods for Identifying Cross-Frequency Couplings in Human EEG

**DOI:** 10.1371/journal.pone.0060513

**Published:** 2013-04-03

**Authors:** Jérôme Van Zaen, Micah M. Murray, Reto A. Meuli, Jean-Marc Vesin

**Affiliations:** 1 Applied Signal Processing Group, Swiss Federal Institute of Technology, Lausanne, Switzerland; 2 EEG Brain Mapping Core, Center for Biomedical Imaging of Lausanne and Geneva, Lausanne, Switzerland; 3 Neuropsychology and Neurorehabilitation Service, University Hospital Center and University of Lausanne, Lausanne, Switzerland; 4 Radiology Service, University Hospital Center and University of Lausanne, Lausanne, Switzerland; University of British Columbia, Canada

## Abstract

Oscillations have been increasingly recognized as a core property of neural responses that contribute to spontaneous, induced, and evoked activities within and between individual neurons and neural ensembles. They are considered as a prominent mechanism for information processing within and communication between brain areas. More recently, it has been proposed that interactions between periodic components at different frequencies, known as cross-frequency couplings, may support the integration of neuronal oscillations at different temporal and spatial scales. The present study details methods based on an adaptive frequency tracking approach that improve the quantification and statistical analysis of oscillatory components and cross-frequency couplings. This approach allows for time-varying instantaneous frequency, which is particularly important when measuring phase interactions between components. We compared this adaptive approach to traditional band-pass filters in their measurement of phase-amplitude and phase-phase cross-frequency couplings. Evaluations were performed with synthetic signals and EEG data recorded from healthy humans performing an illusory contour discrimination task. First, the synthetic signals in conjunction with Monte Carlo simulations highlighted two desirable features of the proposed algorithm vs. classical filter-bank approaches: resilience to broad-band noise and oscillatory interference. Second, the analyses with real EEG signals revealed statistically more robust effects (i.e. improved sensitivity) when using an adaptive frequency tracking framework, particularly when identifying phase-amplitude couplings. This was further confirmed after generating surrogate signals from the real EEG data. Adaptive frequency tracking appears to improve the measurements of cross-frequency couplings through precise extraction of neuronal oscillations.

## Introduction

Oscillatory activity is a key component of brain dynamics and has increasingly been the focus of neuroscientific research. Neuronal oscillations have been considered a possible mechanism through which internal states exercise top-down influences on stimulus processing to impact perception [1], [2]. In particular, the phase synchronization of oscillatory components seems to be relevant for many cognitive processes [3]. Different models have been proposed for explaining the role of neural synchronization. For instance, the “communication through coherence” model [4] suggests that phase synchronization is a binding mechanism through which communication between different cortical areas is established. Another model proposes that phase synchronization facilitates neuronal plasticity [5]. Other studies [6], [7] consider that large-scale integration of perception into a unified representation is supported by neural synchronization. Therefore, synchronization of neuronal oscillations is considered a key mechanism for solving the problem of binding multiple and/or distributed representations. Moreover, this mechanism not only encompasses interactions between different cortical areas but also interactions between classical neuronal frequency bands; so-called cross-frequency couplings [8]. These cross-frequency couplings have been proposed as a framework for unifying the neuronal oscillations at different temporal and spatial scales [9]. The importance of these coupling processes have been demonstrated in recent studies of motor, sensory and cognitive tasks (e.g. [10]–[17]).

The reliability of methods for identifying these interactions across frequency bands can be examined using the well-known illusory contour (IC) stimuli [18]. Investigators have considered this paradigm as exemplary of the binding problem because physically absent borders of an object must be “filled-in” (at least perceptually if not also neurophysiologically) between inducers. One consistent observation is increased gamma power for IC vs. control stimuli (e.g. [19]–[21]). Another highly replicable finding is stronger global field power in the ERP to the presence vs. absence of ICs (e.g. [22]–[26]). The case of IC processing thus exemplifies a situation where the relationship between effects observed using analyses of event-related potentials (ERPs; which are heavily influenced by lower-frequency oscillations below ∼25 Hz) and those obtained using time-frequency analyses (which typically focus on higher-frequency oscillations above ∼25 Hz) remains to be detailed and ultimately conjoined (e.g. [27]). Moreover and despite being the subject of neuroscientific investigation spanning several decades in both humans and animal models, controversy persists regarding whether ICs are the result of bottom-up vs. top-down mechanisms (e.g. [26]). These kinds of results highlight the need for signal processing methods that can detail relationships between extracted features in a statistically sound manner.

Neural synchronization underlying cross-frequency couplings has been studied with a large number of different tools. In particular, methods based on phase information, such as phase locking value [28], [29], have been applied to EEG data. Moreover, it has been shown recently that phase can encode more information than power [30], and thus such methods are well-suited to analyze cross-frequency interactions. The phase information is typically extracted with the widely-used Hilbert transform [31], but it should be considered with caution. The extracted phase is guaranteed to be physically meaningful only for narrow-band signals [32], and thus phase interpretation is problematic for broad-band signals. It should be noted that this interpretation problem arises with any technique for phase extraction. Consequently, the phase locking value is sensitive to broad-band interference [33]. A straightforward solution to this problem consists of adding a pre-processing step that separates EEG data into various narrow frequency bands with band-pass filters or wavelet analysis. Although this filter-bank approach can lead to more reliable analyses of cross-frequency couplings [10], it has a major disadvantage. The specifications of the filters (e.g. cut-off frequencies, attenuation, etc.) are predefined without taking into account the dynamics of the EEG signal under investigation. Therefore, an oscillatory component whose instantaneous frequency crosses the limit between two bands would be considered as two different oscillations occurring successively. In such cases it would be preferable to apply adaptive methods that can track a periodic component with a time-varying instantaneous frequency in a continuous manner. We recently proposed such a technique [34], [35] in which a time-varying band-pass filter is adapted over time in order to extract an oscillation and its instantaneous frequency.

**Figure 1:**
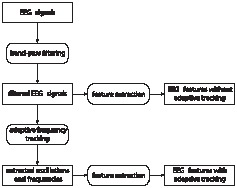
Processing steps used for analyzing the EEG data. The same features were extracted before and after frequency tracking in order to assess the usefulness of the proposed adaptive algorithm.

In this study, we used such adaptive filters for analyzing the evolution of phase-amplitude and phase-phase couplings in response to the presence vs. absence of ICs. Although we still relied on predefined band-pass filters for separating the signals into various frequency bands, we chose wider filters than the ones typically used for processing EEG data. The following step was to retrieve the main oscillatory component and its instantaneous frequency in each band with our adaptive frequency tracking scheme. Thus, we obtained narrow band signals from which we could precisely extract the phase information, which, in turn, was used for measuring phase-amplitude and phase-phase coupling strength over time. The complete procedure is summarized in Figure 1. In more detail, we tested three aspects of cross-frequency couplings during IC perception. First, we checked that stimuli with and without IC elicited a change in terms of coupling strength by using surrogate stationary signals generated from the original EEG data. This analysis assessed if the two types of stimuli caused a response before conducting further tests. Second, we contrasted the responses to stimuli with IC to the ones without such contours. One goal of this study was to determine if ICs elicited specific changes in terms of coupling strength. Last, we compared the results obtained with and without adaptive frequency tracking in order to highlight the value of our algorithm for precisely extracting the phase information and measuring cross-frequency couplings. Synthetic signals were also used for showing the advantages of frequency tracking. Specifically, classical approaches were compared to tracking for measuring cross-frequency couplings with such signals. Compared to our previous study which presented the algorithm for adaptive frequency tracking and possible applications in EEG data processing [35], the present study constitutes a more thorough analysis at a group-level as well as dynamic analysis of instantaneous frequency and cross-frequency couplings following IC presentation.

## Materials and Methods

### Experimental Setup

The present study is a re-analysis of a subset of data appearing in a previously published study that focused on broad-band ERPs in response to IC stimuli [22]. Full details regarding the experimental setup can be obtained from the original study. Here, we provide only the essentials.

The participants in the present study included nine healthy adults (seven men and two women), aged 22–47 years (mean ± SD = 34±10 years). Seven of the participants were right-handed and two left-handed according to Edinburgh inventory [36]. All procedures involved in the original data acquisition were approved by the Institutional Review Board of the Nathan Kline Institute for Psychiatric Research. All participants provided written informed consent.

**Figure 2:**
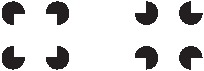
Experimental conditions. IC condition (left), NC condition (right).

Participants viewed arrays of “pac-men” inducers presented in either of two orientations. In the illusory contour (IC) condition, the inducers were turned in order to produce the illusory perception of a simple geometric shape. On the contrary, in the no contour (NC) condition, the inducers were rotated 180° outwards; this prevented any illusory perception with the same luminance and contrast. Examples of the two conditions are shown in Figure 2. Each stimulus appeared for 500 ms, followed for 1000 ms by a blank screen. A Yes/No response prompt appeared then and remained visible until a decision was made, allowing subjects to control stimulus presentation. Subjects pressed a button for “Yes” when they perceived an illusory shape and a second button for “No” when it was not the case. The response was followed by a blank screen for 1000 ms. The response prompt was used to clearly separate the sensory response from the motor response.

### Data Acquisition and Pre-processing

Continuous EEG was recorded through a Neuroscan Synamp from 64 scalp electrodes (impedances ≤5 kΩ), referenced to the nose, band-pass filtered from 0.05 Hz to 100 Hz, and sampled at 500 Hz. Trials for each subject and condition were visually selected with the Cartool software by Denis Brunet (http://sites.google.com/site/fbmlab/cartool/) [37]. A threshold of ±80 µV for artifact rejection was used. Each trial represented 2000 ms of EEG data, with stimulus onset after 500 ms. There were an average (± SD) of 300 (±57) EEG trials from the IC condition and 295 (±43) trials from the NC condition included in the analyses. Once the trials were extracted, all further processing was performed in Matlab.

First, the EEG signals recorded from a cluster of five electrodes (P2, P4, P6, PO4 and PO6) were selected. This selection was based on the right-lateralized posterior scalp distribution of the ERP difference between IC and NC conditions (cf. Figure 3 in [22]). Then, signals from these electrodes were re-sampled from 500 Hz to 250 Hz and the power line interference at 60 Hz was canceled with a narrow notch filter. (The original recordings were performed at the Nathan Kline Institute for Psychiatric Research in Orangeburg, New York, USA.) The spatial mean of the five electrodes was computed in order to obtain a slightly more global view. Due to the proximity of the selected electrodes, the corresponding EEG signals were almost identical. The signals obtained after spatial averaging were then filtered in the following frequency bands: 1–4 Hz, 4–8 Hz, 8–12 Hz, 15–25 Hz, 35–45 Hz, 45–55 Hz, 55–65 Hz and 65–75 Hz. It is important to mention that the signals were filtered in both forward and reverse directions in order to achieve zero-phase distortion [38]. Consequently, the samples at the beginning and at the end should be considered with caution as they were susceptible to transients and border effects. Once the band-pass signals were obtained, we applied the adaptive frequency tracking scheme that we recently proposed [34], [35] in order to extract the main oscillation in each band as well as its estimated instantaneous frequency.

**Figure 3:**
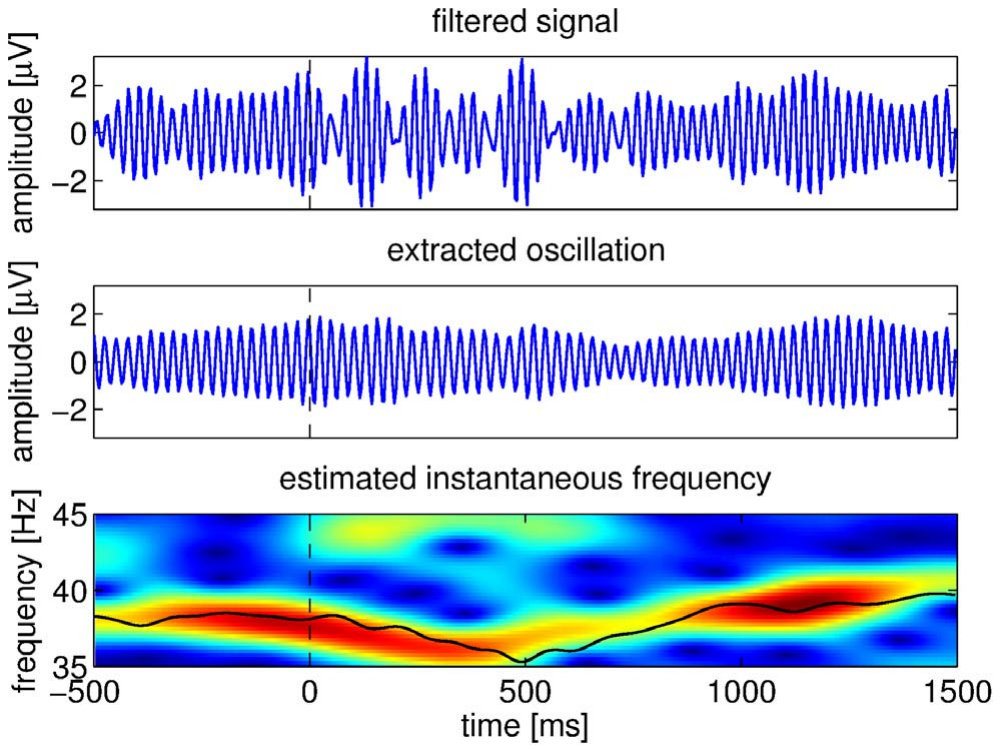
Pre-processing steps. EEG signal filtered in the 35–45 Hz band (top), oscillation extracted with the SFT (middle), and its estimated instantaneous frequency (bottom). The estimated frequency is plotted on top of the short-time Fourier transform of the EEG signal. The vertical dashed lines denote stimulus onset.

This adaptive scheme is called the single frequency tracker (SFT). It has been developed in the complex-valued signal framework, and thus it must be applied to the analytic representation of the input signal. This representation can be obtained with the discrete Hilbert transform [39]. A real output signal can always be recovered by keeping only the real part. The SFT is based on an adaptive band-pass filter whose transfer function is defined as follows,
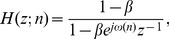
(1)


where 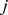 is the imaginary unit, 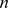 denotes the discrete time, 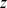 is the variable from the Z-transform, 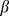 (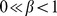) determines the bandwidth and 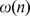 is the instantaneous frequency estimate that controls the central frequency. This filter has unit gain and zero phase at 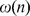 which is of the utmost importance for measuring coupling based on phase information. The frequency estimate is computed recursively using the following equations:
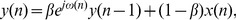
(2)

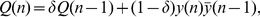
(3)

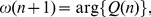
(4)


where 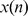 and 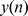 are the input and output signals, 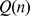 is an internal variable, 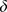 (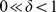) is a forgetting factor that controls the convergence rate and the upper bar denotes complex conjugation. This adaptive scheme extracts the main oscillation in a given signal and estimates its instantaneous frequency. In this study, the input signal was one of the band-pass filtered EEG signals, while the output signal was the corresponding extracted neuronal oscillation.

To summarize the pre-processing, we computed the spatial average of a cluster of five electrodes, then the signals were separated into various frequency bands with fixed band-pass filters, and finally we applied the SFT in order to extract the main oscillation and its instantaneous frequency in each band with a narrow time-varying band-pass filter. We selected the following values for the parameters of the tracking scheme: 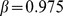 (this corresponds approximately to a 3 dB bandwidth of 2 Hz), 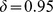, and the initial frequency was set to the center of the considered frequency band. The selected values for 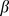 and 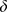 offered a good trade-off between adaptation speed and accurate oscillation extraction. It is also worth mentioning that small variations around these values should affect the results only marginally. For proper initialization of the internal variable, we applied the SFT to longer signals obtained by adding the mirrored first 500 ms at the beginning. The input and outputs of the SFT are illustrated in Figure 3 with an EEG signal filtered in the 35–45 Hz band. It is possible to see that the tracking is not immediate, because the adaptation introduces a slight delay. Finally, we obtained one oscillatory component and its estimated instantaneous frequency for each frequency band.

**Figure 4:**
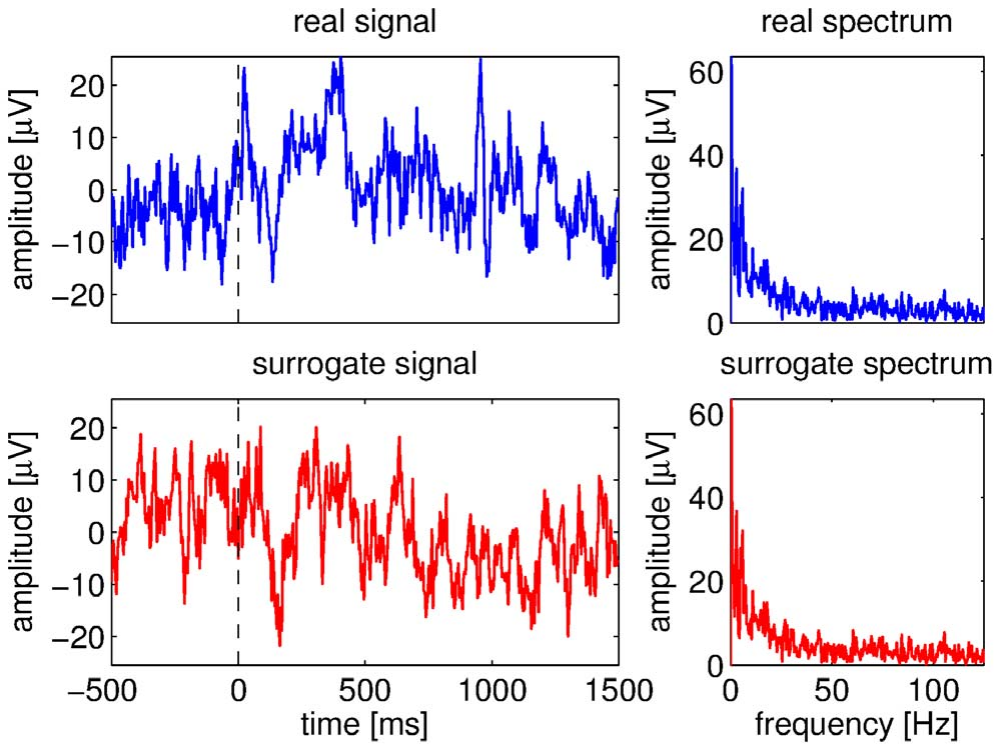
Original and surrogate EEG signals. Real EEG signal (top left), surrogate signal (bottom left), amplitude spectrum of the real signal (top right), amplitude spectrum of the surrogate signal (bottom right). Vertical dashed lines denote stimulus onset.

In order to assess the presence or absence of phenomena, surrogate EEG signals were generated from the original data [40], [41]. This was done as follows: (1) an EEG signal was transformed into the frequency domain with the discrete Fourier transform, (2) then the amplitudes were kept but the phases were randomized (random variables drawn from a uniform distribution between 0 and 2π), (3) finally the modified signal was transformed back into the time domain. The phase randomization destroys the structure in the input signal and yields a more stationary output. However, the surrogate signal shares some properties with the original one, such as probability density function and autocorrelation. Thus, they have also the same power spectral density. This surrogate approach can help to highlight non-stationary effects, like stimulus-locked responses. This operation was repeated in order to obtain one surrogate signal for each EEG signal. Then, the same pre-processing was applied to the surrogate signals (fixed band-pass filtering and adaptive frequency tracking). Therefore, we obtained two datasets: a real dataset (corresponding to the real EEG signals) and a surrogate dataset. An example of surrogate signal is shown in Figure 4. One can observe that although the real and surrogate signals are different, they have the same amplitude spectrum.

### Features

Once the pre-processing was applied to all EEG signals (real and surrogate ones), three different features were investigated. They were used for highlighting differences between real and surrogate datasets as well as between the two conditions used in the experiment (IC and NC). They also permitted to assess the usefulness of the SFT, as the same features were computed before and after frequency tracking. The features were computed on sliding windows of length 300 ms which offered a good trade-off between temporal resolution and estimation accuracy. The time shift between successive windows was set to 10 ms. Sliding windows were used in order to visualize the evolution of the features over time. The complete procedure for extracting the features from the spatial-averaged EEG signals is depicted in Figure 1. We focused on three features: the mean instantaneous frequency estimated by the SFT, the phase-amplitude and phase-phase couplings. All details regarding the computation of these figures are provided in the following sections.

#### Mean Frequency

The first feature that we considered was the mean estimated instantaneous frequency, based on the estimate provided by the SFT. Although we mostly focused on cross-frequency couplings, the mean frequency was also investigated as it was readily available thanks to the SFT. Also, this kind of feature was rarely analyzed in this context. It is important to notice that this feature could not be computed without adaptive frequency tracking.

#### Phase-amplitude Couplings

Phase-amplitude couplings were measured with the phase locking value (PLV) [29]. The PLV is computed using the phase of the low frequency component 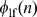 and the phase of the amplitude of the high frequency component 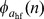:
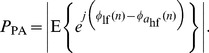
(5)


The phases and amplitudes were extracted with the Hilbert transform. The PLV takes a value of one for perfectly synchronized signals and zero when there is no synchronization. In practice, the expectation was replaced by the sample mean.

#### Phase-phase Couplings

Phase-phase couplings were also measured with the PLV. However, this measure was slightly modified in order to take into account oscillatory components with different frequencies [28]. It is defined as follows for measuring phase-phase couplings:
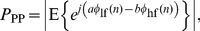
(6)


where 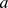 and 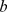 are coupling coefficients. The following values for 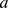
∶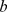 were considered: 4∶3, 3∶2, 2∶1, 3∶1, 4∶1, 5∶1, 6∶1, 7∶1, 8∶1 and 9∶1. Only the PLVs for which the frequency bands and coupling coefficients made sense were computed (i.e. bands that overlapped once multiplied by the corresponding coupling coefficients). For instance, when measuring the phase-phase couplings between 1–4 Hz and 35–45 Hz components, the coupling coefficients 2∶1 were discarded. Indeed, multiplying the limits of each band with the corresponding coefficient yields 2–8 Hz and 35–45 Hz which do not overlap. As for the phase-amplitude couplings, the expectation was replaced by the sample mean.

#### Statistical Analysis

Once the three features were computed over all sliding windows, we performed statistical tests in order to display significant differences between real and surrogate datasets or IC and NC conditions. However, before applying the tests, the features were transformed into approximately Gaussian variables whenever necessary. This was not needed for the mean frequency due to the central limit effect [42]. By contrast, the PLV values for both phase-amplitude and phase-phase couplings were transformed into approximately Gaussian random variables with an arcsine transform (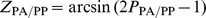) [43]. Finally, analysis of variance (ANOVA) was performed with “subject” as a random effect and “dataset” or “condition” as a fixed effect for the 101 windows whose centers were located in the interval 0–1000 ms following stimulus presentation. We considered only the p-value for the fixed effect. As many tests were performed for each feature which could lead to several type I errors, an effect was declared significant only when the ANOVA yielded a p-value below 5% for some number of successive windows. Furthermore, we used permutation tests [44] to compute a lower bound for this number of successive significant windows in order to achieve a final p-value below 5%. These tests were performed by repeating 1000 times the ANOVA with randomly permuted dataset or condition memberships. In other words, the features computed on sliding windows were randomly reassigned to either of the datasets or conditions while keeping the true subjects' assignments and the natural temporal order of the windows so as to preserve the correlation structure. The p-values for all windows were then computed with the ANOVA, and the maximum number of successive significant windows (p<0.05) was evaluated for the 1000 repetitions. Thus, we could estimate the distribution of the maximum number of successive significant windows under the hypothesis of no difference between the datasets or conditions. And therefore, we could compute an estimate of the probability of observing a number of successive significant windows equal to or greater than the one obtained with the true assignment of datasets or conditions when assuming no difference between these datasets or conditions. This probability estimate is in fact the p-value for the number of successive significant windows for the feature under investigation. In practice, we declared a difference significant only when this p-value was below 5% and the ANOVA rejected the null hypothesis for at least 4 successive windows. The latter condition ensured that the observed difference was not only punctual.

### Synthetic Signals

The usefulness of the SFT for measuring cross-frequency couplings was also evaluated with synthetic signals and Monte Carlo simulations. We considered two cases: a basic case with sinusoids with additive noise and a more complex case in which synthetic signals were generated in order to mimic a real EEG signal. In the first case, the goal was to measure the phase-phase couplings with the PLV between two simple signals. The input signals were defined as two sinusoids at normalized frequencies 0.05 and 0.35 with uniformly distributed phases embedded in independent white Gaussian noises:
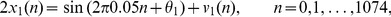
(7a)

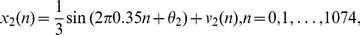
(7b)


where 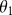 and 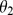 were the random phase terms uniformly distributed between 0 and 2π, and 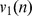 and 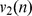 were the additive white Gaussian noises. The random phase terms and noises were mutually independent. The 1/3 factor was used to take into account the power decrease in higher frequencies in EEG data. Then, the SFT was applied for extracting the oscillatory component in each signal. The parameters of the adaptive algorithm were set to 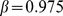 and 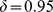. The phases of input and output signals were extracted with the discrete Hilbert transform. The first and last 500 samples were then discarded in order to avoid any border effect. This yielded 75-samples phase signals whose length corresponded to the length of the 300-ms windows at 250 Hz used in the EEG analysis. Finally, the PLV was computed with coupling coefficients set to 7∶1 (ratio of the frequencies: 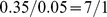) for the phase signals obtained with and without frequency tracking. The PLV mean and standard deviation were estimated with 10,000 Monte Carlo simulations for the two approaches. In each repetition, new random values for 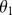, 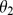, 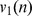 and 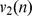 were generated. Furthermore, this procedure was repeated for SNR values ranging from 0 to 20 dB in 1 dB steps. It is important to note that without noise the PLV for these signals should be equal to one.

In the second case, we generated two 500-samples signals mimicking the outputs of the band-pass filters used when analyzing the real EEG data. The sampling frequency for these two synthetic signals was set to 250 Hz which corresponded to a duration of 2000 ms. The first signal was defined as
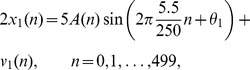
(8a)

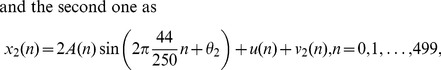
(8b)


where 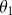 and 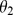 were random phase terms uniformly distributed between 0 and 2π, 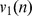 and 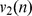 were additive white Gaussian noises with variances 25 and 4 respectively, 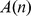 was the time-varying amplitude of the sinusoids and 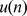 was a periodic interference at 36 Hz. They were set to
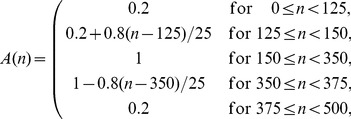
(9)


and 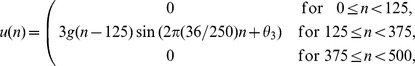
(10)


where 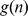 is a 250-samples Hann window [38] and 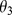 is a random phase term uniformly distributed between 0 and 2π. As in the first case, the phase terms and noises were mutually independent. The first signal was then filtered in the 4–8 Hz band and the second one in the 35–45 Hz band with the same fixed band-pass filters used before. All the parameters were chosen in order to generate synthetic signals inspired by a real EEG signal with stable oscillatory components at 5.5 and 44 Hz and a short periodic interference at 36 Hz. The SFT was applied to both signals for extracting the main periodic components with the same parameters and mirroring procedure as for the real EEG data. The phase-phase couplings were then measured by computing the PLV with coupling coefficients 8∶1 over sliding windows of length 300 ms shifted by 10 ms. The results obtained with and without tracking were averaged over 10,000 realizations of the random phases and noises. In this synthetic example, there should be an increase in coupling strength when *n* is between 150 and 350 samples (600–1400 ms).

## Results

### Synthetic Signals

**Figure 5:**
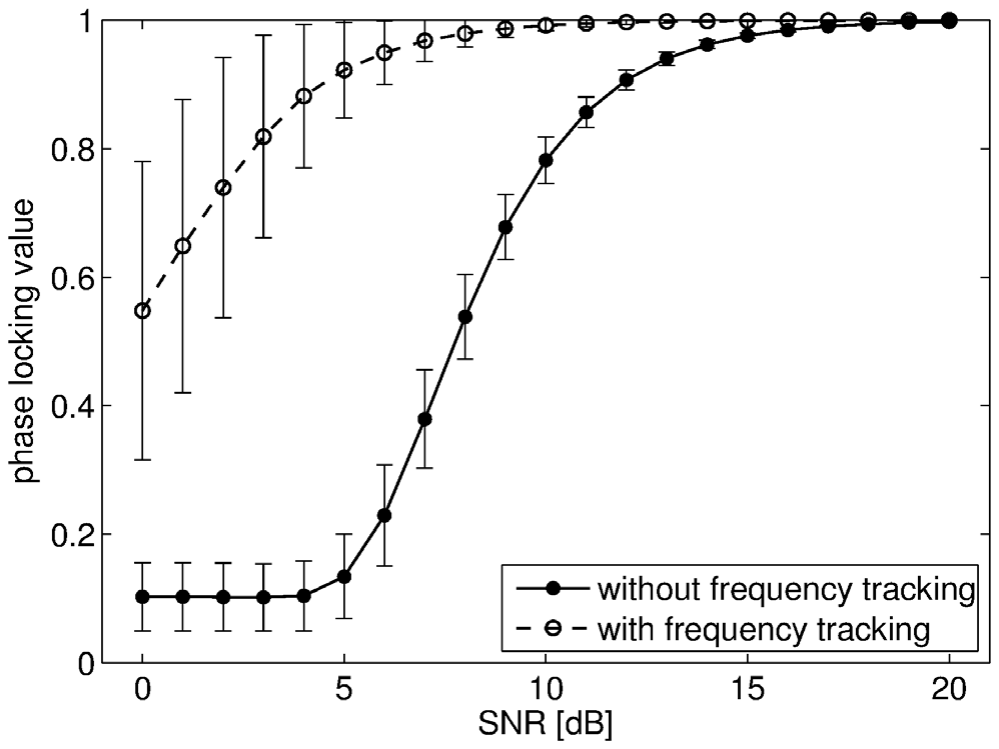
PLV values for the first synthetic example. The mean PLV values obtained with and without adaptive frequency tracking are shown with corresponding error bars for SNR values ranging from 0 to 20 dB in 1 dB steps.

**Figure 6:**
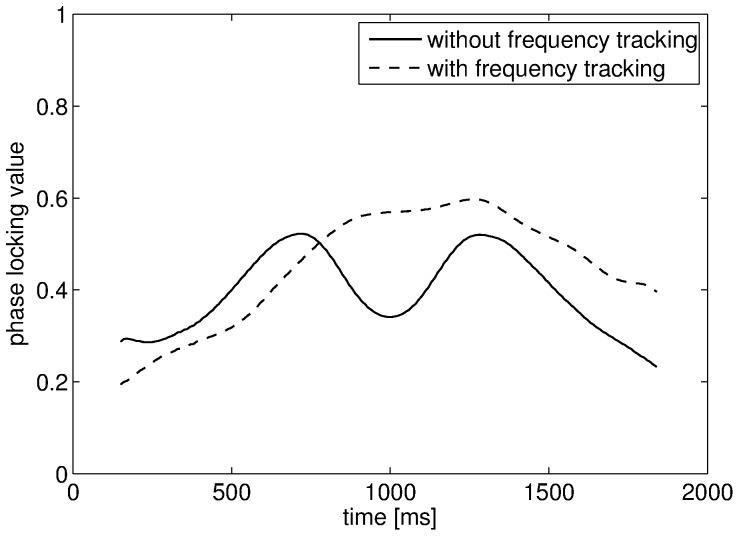
PLV values for the second synthetic example. The mean PLV values were measured over 300-ms sliding windows.

Before presenting the results obtained with the signals recorded during the IC experiment, we present the outcomes of the Monte Carlo simulations with synthetic signals. The mean PLVs with error bars obtained with the first set of signals are shown in Figure 5 for all tested SNR values. Without noise the PLV should be equal to one in this scenario as the two oscillatory components were perfectly synchronized with 7∶1 coefficients. This was indeed the case for very low noise levels. However, without adaptive tracking, the mean PLV quickly decreased as the noise variance increased. This decrease was quite severe even for moderate noise levels. By contrast, the SFT led to PLV values that were much more resilient to noise, at the cost of ed estimation variance however. Nevertheless, the SFT increased the overall performance of the PLV for measuring phase-phase couplings with these synthetic signals. Indeed, although the PLV variance was higher with tracking, the mean PLV obtained without tracking reached its minimal value for SNR values below 5 dB. In the second case, where synthetic signals were generated so as to imitate real EEG data, the SFT also proved to be helpful for measuring phase-phase couplings. These signals contained two perfectly synchronized sinusoids with 8∶1 coefficients embedded in noise. An interfering periodic component active during a short duration was also present in the high-frequency signal. The two sinusoids had time-varying amplitudes that reached their maximal values in the interval 600–1400 ms. And thus, the PLV computed over 300-ms sliding windows shifted by 10 ms should increase in this interval. The results averaged over 10,000 Monte Carlo simulations are shown in Figure 6 with and without frequency tracking. Without tracking, the PLV increased in the beginning of the interval as expected, but it was then completely disrupted by the interfering oscillation. In the end of the interval, it increased again as the amplitude of the interference dropped. On the contrary, the PLV values obtained with the SFT were higher during the whole duration of the interval, except for an adaptation delay (∼150 ms). Therefore, with these synthetic signals, meaningful phase information could be extracted thanks to the proposed adaptive algorithm which led to robust PLV values. In particular, the first example showed its tolerance to broad-band noise, while the second one showed its resilience to interfering oscillations.

### Mean Frequency

**Figure 7:**
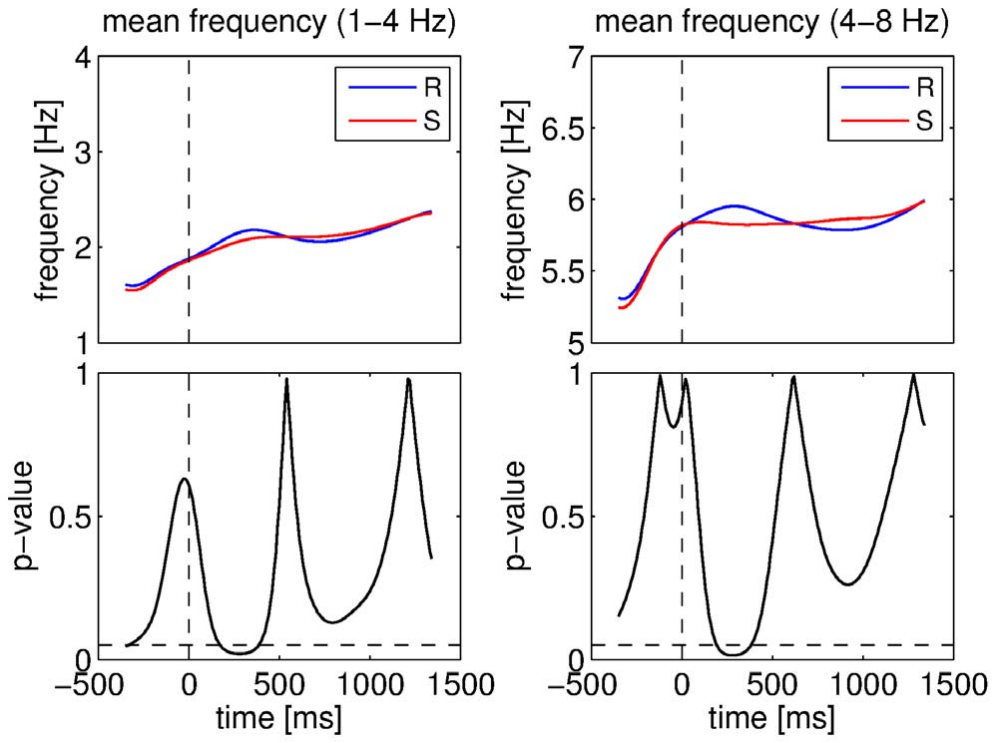
Comparisons between datasets for the mean estimated frequency. Mean estimated frequency for the 1–4 Hz component for real (R) and surrogate (S) datasets (top left), ANOVA p-values for the 1–4 Hz component (bottom left), mean estimated frequency for the 4–8 Hz component for real and surrogate datasets (top right), ANOVA p-values for the 4–8 Hz component (bottom right). Vertical dashed lines denote stimulus onset and horizontal ones denote the 5% significance level.

The comparisons between the real and surrogate datasets yielded significant differences in terms of mean frequency for the 1–4 Hz component in the interval 180–380 ms (21 successive windows, permutation test: p<0.001) and the 4–8 Hz component in the interval 200–380 ms (19 successive windows, permutation test: p<0.001). In both cases, the estimated instantaneous frequency was higher for the real data than for the surrogate ones. This is illustrated in Figure 7. It seems that the mean frequency of the main oscillatory component in the band 1–4 Hz increased smoothly following stimulus presentation for the two datasets. However, this frequency increase was more important in the real data. The phenomenon was slightly different for the 4–8 Hz band. Indeed, in this case, the main frequency remained almost constant for the surrogate dataset. By contrast, an increase in mean frequency after stimulus presentation for the real dataset caused the significant difference. There was no other significant difference between the two datasets for this feature.

**Figure 8:**
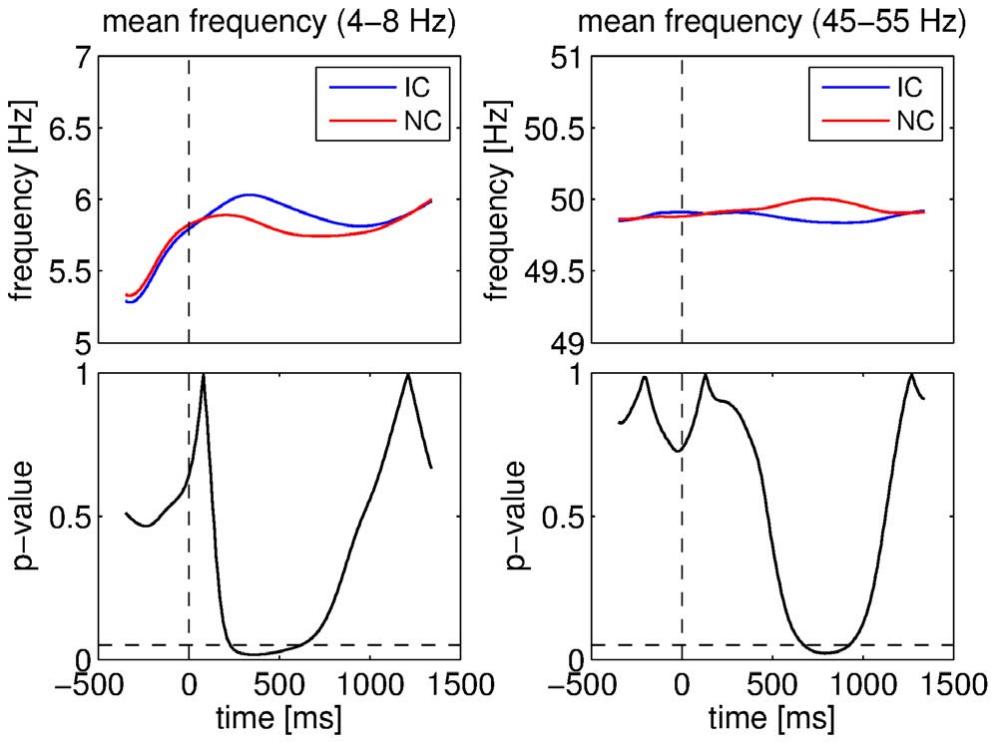
Comparisons between conditions for the mean estimated frequency. Mean estimated frequency for the 4–8 Hz component for IC and NC conditions (top left), ANOVA p-values for the 4–8 Hz component (bottom left), mean estimated frequency for the 45–55 Hz component for IC and NC conditions (top right), ANOVA p-values for the 45–55 Hz component (bottom right). Vertical dashed lines denote stimulus onset and horizontal ones denote the 5% significance level.

As significant differences in mean frequency were observed between the real and surrogate datasets, we also compared the IC and NC conditions with respect to this feature. We obtained significant differences between the two conditions for the instantaneous frequency of the components in the 4–8 Hz and 45–55 Hz bands. These two comparisons are shown in Figure 8. The frequency was significantly higher for IC than for NC for the 4–8 Hz component in the interval 230–610 ms (39 successive windows, permutation test: p<0.001). The frequency increase following stimulus presentation was more pronounced and lasted longer for IC than for NC. By contrast, IC yielded a lower mean frequency than NC for the 45–55 Hz component. This difference was significant in the interval 680–920 ms (25 successive windows, permutation test: p<0.05). In this case, the frequency decreased more than 500 ms after stimulus for IC while it increased for NC. No other significant difference between the conditions was observed in terms of frequency.

### Phase-amplitude Couplings

**Figure 9:**
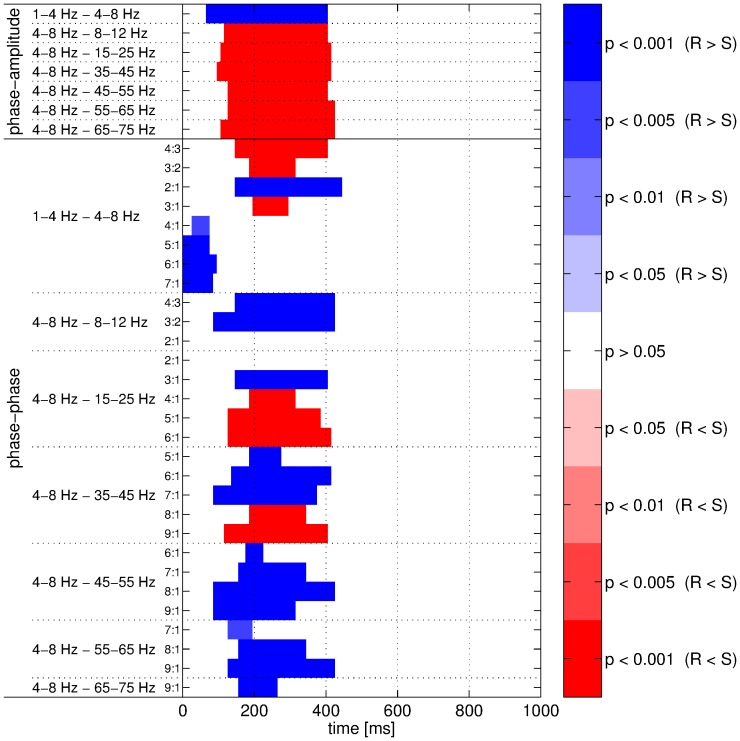
Comparisons between datasets for cross-frequency couplings. The phase-amplitude (top rows) and phase-phase (bottom rows) couplings were measured with adaptive frequency tracking. Significant intervals are shown in blue (respectively in red) when the coupling strength was higher for the real (R) (respectively surrogate (S)) dataset. Color intensity denotes significance level of the corresponding permutation test.

**Figure 10:**
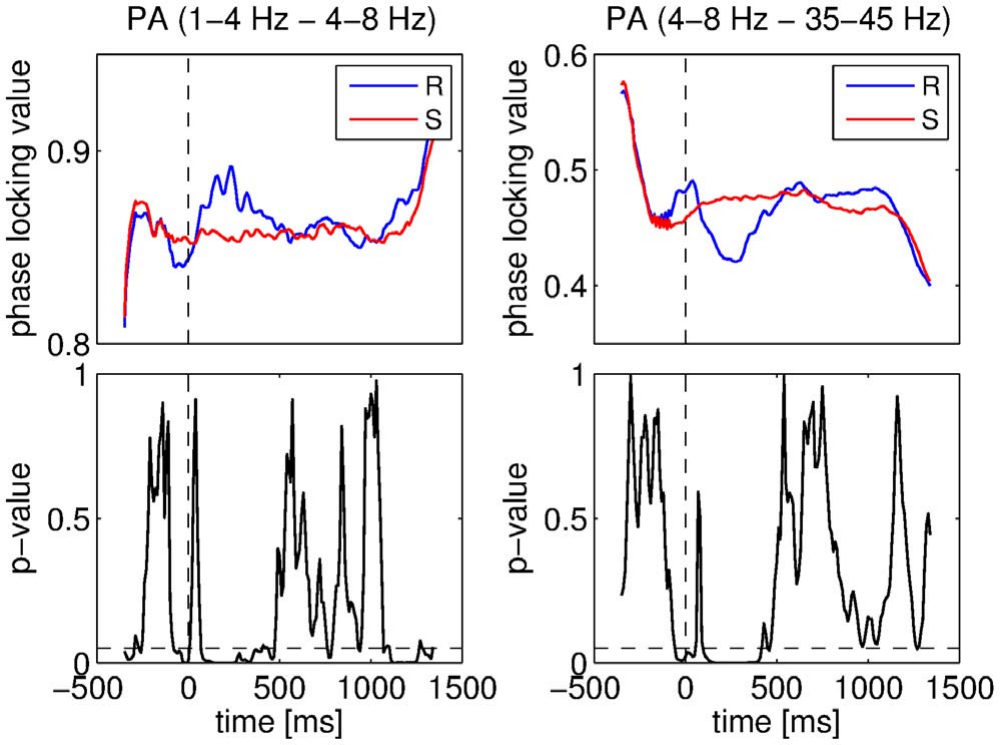
Comparisons between datasets for the phase-amplitude (PA) couplings. Coupling strength for real (R) and surrogate (S) datasets was measured using the PLV. Mean PLV for the 1–4 Hz and 4–8 Hz components for real and surrogate datasets (top left), ANOVA p-values for these components (bottom left), mean PLV for the 4–8 Hz and 35–45 Hz components for real and surrogate datasets (top right), ANOVA p-values for these components (bottom right). Vertical dashed lines denote stimulus onset and horizontal ones denote the 5% significance level.

Several combinations of components yielded significant differences after stimulus onset in phase-amplitude couplings when comparing real and surrogate datasets. Most of the significant results were obtained when the 4–8 Hz component was involved. A significant decrease in coupling strength was observed after stimulus presentation for the real dataset compared to the surrogate one when the 4–8 Hz component was considered as the low frequency component. By contrast, an increase was obtained with the components from the 1–4 Hz and 4–8 Hz bands. All these results involving the 4–8 Hz component are summarized in Figure 9. Furthermore, two examples of comparisons between the real and surrogate datasets in terms of phase-amplitude couplings are shown in Figure 10 for the combinations 1–4 Hz and 4–8 Hz as well as 4–8 Hz and 35–45 Hz. Other significant results were obtained with the following pairs of components: 1–4 Hz and 8–12 Hz in the interval 170–320 ms (real > surrogate, 16 successive windows, permutation test: p<0.001), 1–4 Hz and 15–25 Hz in the interval 190–420 ms (real > surrogate, 24 successive windows, permutation test: p<0.001), 1–4 Hz and 55–65 Hz in the interval 220–340 ms (real > surrogate, 13 successive windows, permutation test: p<0.01), 8–12 Hz and 15–25 Hz in the interval 90–280 ms (real < surrogate, 20 successive windows, permutation test: p<0.01), and 8–12 Hz and 35–45 Hz in the interval 210–240 ms (real < surrogate, 4 successive windows, permutation test: p<0.05).

**Figure 11:**
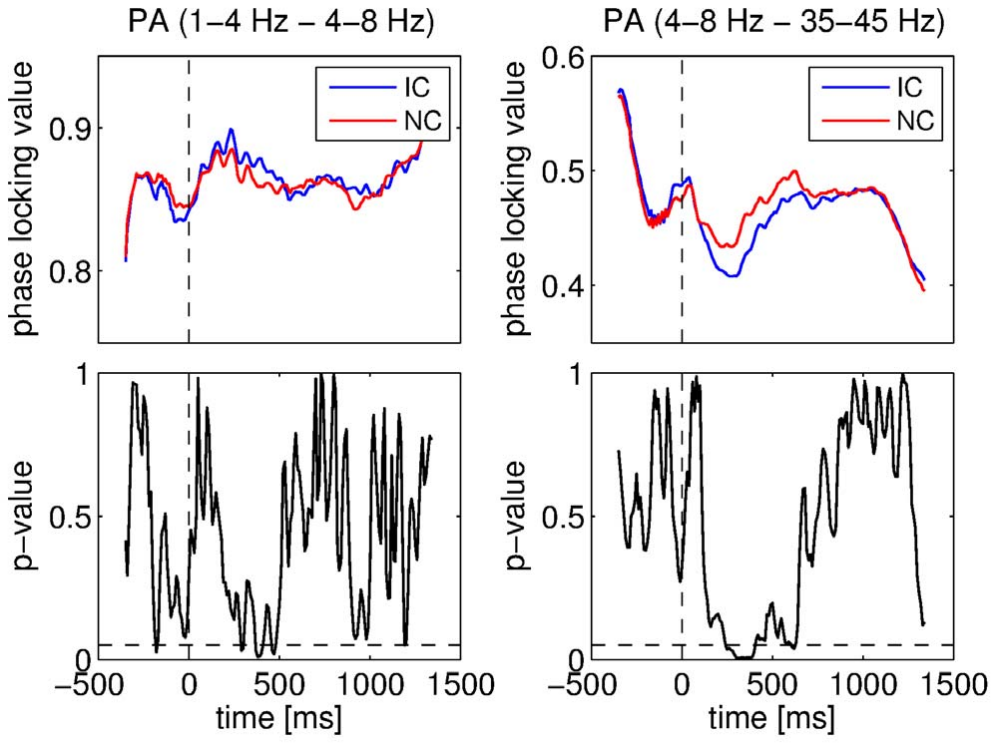
Comparisons between conditions for the phase-amplitude (PA) couplings. Coupling strength for IC and NC conditions was measured using the PLV. Mean PLV for the 1–4 Hz and 4–8 Hz components for IC and NC conditions (top left), ANOVA p-values for these components (bottom left), mean PLV for the 4–8 Hz and 35–45 Hz components for IC and NC conditions (top right), ANOVA p-values for these components (bottom right). Vertical dashed lines denote stimulus onset and horizontal ones denote the 5% significance level.

**Figure 12:**
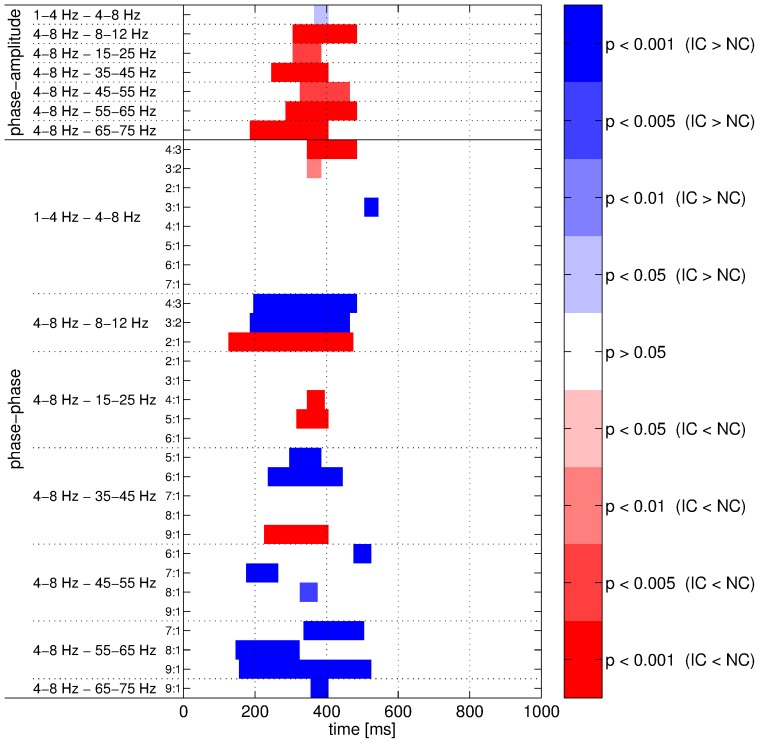
Comparisons between conditions for cross-frequency couplings. The phase-amplitude (top rows) and phase-phase (bottom rows) couplings were measured with adaptive frequency tracking. Significant intervals are shown in blue (respectively in red) when the coupling strength was higher for the IC (respectively NC) condition. Color intensity denotes significance level of the corresponding permutation test.

We also compared the strength of phase-amplitude couplings for the IC and NC conditions. All significant differences were found when the 4–8 Hz component was involved. In fact, when the 4–8 Hz component was considered as the low frequency component, the coupling strength was smaller for IC than for NC. We obtained the inverse when the 4–8 Hz component was considered as the high frequency component (this was only the case for the combination 1–4 Hz and 4–8 Hz). An example of this phenomenon is illustrated in Figure 11. It can be seen that the stimulus caused a change in phase-amplitude coupling strength (either an increase or a decrease) and that this change was always more pronounced for the IC than for the NC condition. All the results about the differences between the two conditions for the phase-amplitude couplings involving the 4–8 Hz component are illustrated in Figure 12.

### Phase-phase couplings

**Figure 13:**
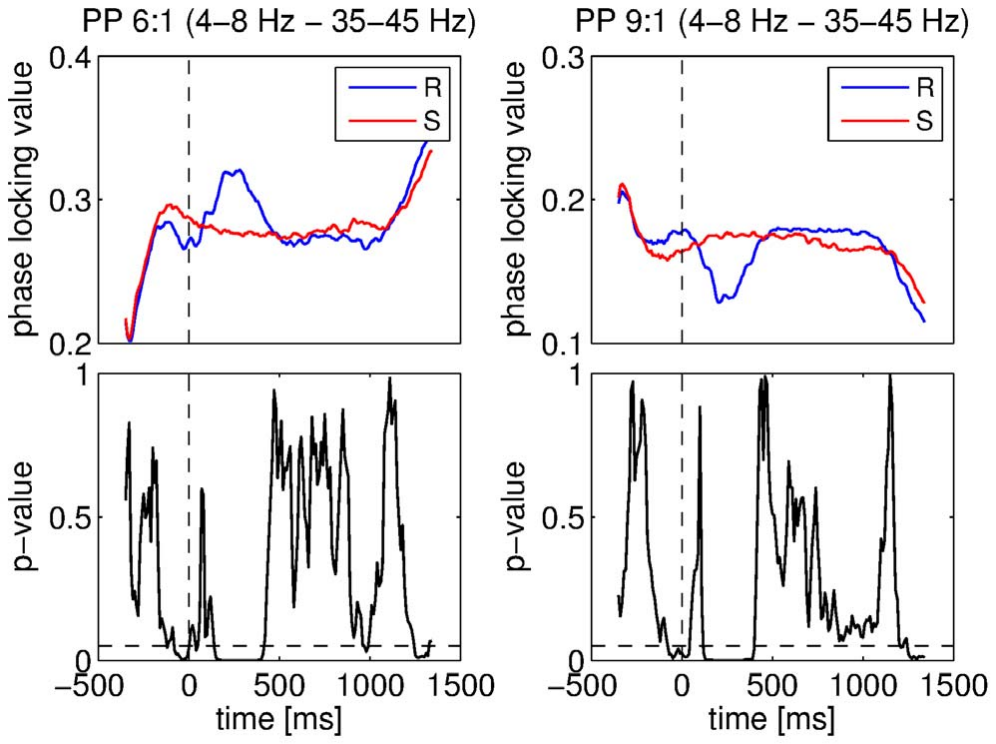
Comparisons between datasets for the phase-phase (PP) couplings. Coupling strength between the 4–8 Hz and 35–45 Hz components for real (R) and surrogate (S) datasets was measured using the PLV. Mean PLV with coupling coefficients set to 6∶1 for real and surrogate datasets (top left), ANOVA p-values for these coefficients (bottom left), mean PLV with coupling coefficients set to 9∶1 for real and surrogate datasets (top right), ANOVA p-values for these coefficients (bottom right). Vertical dashed lines denote stimulus onset and horizontal ones denote the 5% significance level.

The pattern of results was more complex for the phase-phase couplings. Different results were obtained for different coupling coefficients. However, similarly to the phase-amplitude couplings, most of the significant differences were observed when the 4–8 Hz component was considered either as the low- or high-frequency component. When comparing real and surrogate datasets, the dataset yielding higher coupling strength varied depending on the ratio of coupling coefficients. The phase-phase coupling between the 1–4 Hz and 4–8 Hz components were lower for the real dataset than for the surrogate one when a low coefficient ratio was used (4∶3 and 3∶2). On the contrary, when this ratio increased (from 4∶1), the coupling strength was higher for the real signals. For coefficients 2∶1, the coupling strength was higher for the real data while it was lower for coefficients 3∶1. When the 4–8 Hz component was considered as the low frequency component, the coupling strength was higher for low ratios of coefficients. But, as before, the opposite result was observed for higher ratios. An example of this inversion phenomenon is shown in Figure 13 for the 4–8 Hz and 35–45 Hz components with coefficient pairs set to 6∶1 and 9∶1. One can observe that these significant differences were caused by sharp changes (either increase or decrease) in the PLV. All the results of the comparisons between real and surrogate datasets for phase-phase couplings for the 4–8 Hz component are summarized in Figure 9. The permutation tests also identified significant differences between the two datasets for several other combinations of bands. The combinations of bands for which the coupling strength was significantly higher for the real data were the 1–4 Hz and 8–12 Hz bands with coefficients 3∶1, 7∶1, 8∶1 and 9∶1, the 1–4 Hz and 15–25 Hz bands with coefficients 6∶1, the 1–4 Hz and 35–45 Hz bands with coefficients 9∶1, the 8–12 Hz and 15–25 Hz bands with coefficients 2∶1 and 3∶1, the 8–12 Hz and 35–45 Hz bands with coefficients 4∶1, the 8–12 Hz and 45–55 Hz bands with coefficients 6∶1, the 8–12 Hz and 55–65 Hz bands with coefficients 8∶1, the 8–12 Hz and 65–75 Hz bands with coefficients 9∶1, the 15–25 Hz and 35–45 Hz bands with coefficients 2∶1. On the other hand, the combinations of bands showing significantly higher coupling strength for the surrogate datasets were the 1–4 Hz and 8–12 Hz band with coefficients 4∶1 and 5∶1, the 8–12 Hz and 35–45 Hz bands with coefficients 3∶1, the 8–12 Hz and 45–55 Hz bands with coefficients 4∶1 and 5∶1, the 8–12 Hz and 55–65 Hz bands with coefficients 6∶1, the 8–12 Hz and 65–75 Hz bands with coefficients 6∶1 and 7∶1. Consequently, the inversion phenomenon was also observed for other combinations of bands, in particular the ones involving the 8–12 Hz band.

**Figure 14:**
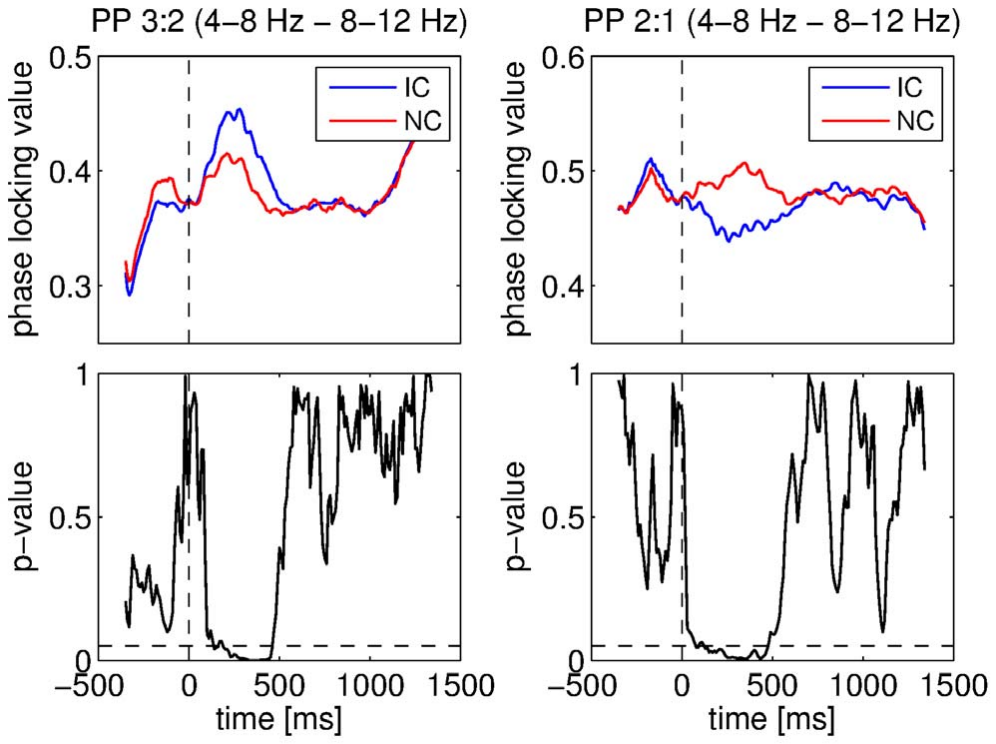
Comparisons between conditions for the phase-phase (PP) couplings. Coupling strength between the 4–8 Hz and 8–12 Hz components was measured using the PLV. Mean PLV with coupling coefficients set to 3∶2 for IC and NC conditions (top left), ANOVA p-values for these coefficients (bottom left), mean PLV with coupling coefficients set to 2∶1 for IC and NC conditions (top right), ANOVA p-values for these coefficients (bottom right). Vertical dashed lines denote stimulus onset and horizontal ones denote the 5% significance level.

A very similar inversion phenomenon depending on coupling coefficients occurred when we compared the IC and NC conditions for the phase-phase couplings. When measuring phase-phase couplings between the 1–4 Hz and 4–8 Hz components, higher coupling strength was observed for NC with low ratios of coefficients (4∶3 and 3∶2). While, for the coefficient 3∶1, IC yielded higher coupling strength. For larger ratios, no clear differences were found for these two bands. When the 4–8 Hz component was considered as the low frequency component, the IC condition led to higher coupling strength compared to the NC condition for low ratios of coefficient pairs. However, as the ratio increased, the condition yielding the higher coupling strength changed to NC. Figure 14 shows an example of this change for the 4–8 Hz and 8–12 Hz components with coefficients set to 3∶2 and 2∶1. We observed this phenomenon for various combinations of frequency bands and coefficient pairs. All the results obtained with the 4–8 Hz component are reported in Figure 12.

### Advantages of Adaptive Frequency Tracking

**Figure 15:**
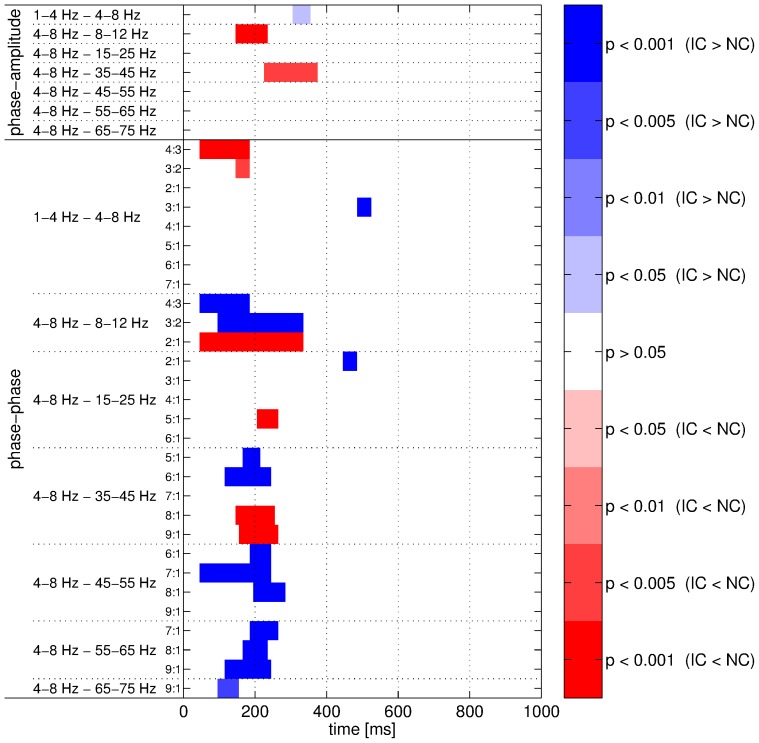
Comparisons between conditions for cross-frequency couplings. The phase-amplitude (top rows) and phase-phase (bottom rows) couplings were measured without adaptive frequency tracking. Significant intervals are shown in blue (respectively in red) when the coupling strength was higher for the IC (respectively NC) condition. Color intensity denotes significance level of the corresponding permutation test.

For assessing the usefulness of the SFT for real EEG data, we compared the two conditions IC and NC when the features were computed without adaptive frequency tracking. In other words, the features were also computed using the output signals of the predefined band-pass filters as shown in Figure 1. Obviously, the mean frequency could not be estimated without frequency tracking as it is specifically an output of the SFT. Nevertheless, the phase-amplitude and phase-phase couplings were measured and the IC and NC conditions were compared with the same test procedure as before. We focused on cross-frequency couplings involving the 4–8 Hz component as they yielded all the significant differences when the SFT was applied. Similarly to Figure 12, the results obtained in this case are documented in Figure 15. Comparing the two figures, one can notice that the results obtained with and without adaptive frequency tracking are very similar and that there is no conflict. However, a more detailed investigation revealed that the differences between IC and NC in terms of coupling strength were, in most cases, more clearly highlighted when using the SFT. In fact, when investigating the differences in cross-frequency couplings involving the 4–8 Hz component, a greater number of successive significant windows was obtained for only seven cases without tracking, including two cases where no significant difference was observed after applying the SFT. For all other cases, the proposed adaptive scheme performed as well as or (more frequently) better than the traditional approach, in terms of number of successive significant windows. It also led to the detection of significant differences between IC and NC which remained unnoticed without tracking in five cases. The usefulness of the SFT is particularly apparent for phase-amplitude couplings. However, it is worth mentioning that the adaptation process in this algorithm introduces a delay. This caused the intervals of significant differences to be shifted in time compared the ones obtained without tracking. A coarse method for comparing the results obtained with and without the SFT is to count the number of successive significant windows in Figures 12 and 15 for phase-amplitude and phase-phase couplings. Thus, for the phase-amplitude couplings, we obtained 102 and 29 windows with and without frequency tracking. These values were 272 and 208 for phase-phase couplings. As mentioned previously, when Figures 12 and 15 are put side-by-side, the significant intervals obtained with the SFT were delayed compared to those obtained without tracking as the adaptation process of the proposed algorithm is not instantaneous. This delay could be quite large depending on the dynamics of the signals under study. Nevertheless, in most cases, it remained reasonable and the intervals overlapped. However, there were a few cases where the delay was quite important (>200 ms). For instance, the significant interval obtained when comparing the phase-phase couplings with 4∶3 coefficients between the 1–4 Hz and 4–8 Hz components occurred 300 ms later with frequency tracking. These long delays, while unusual, could be explained by large non-stationary dynamics in the input signals of the SFT. Indeed, the tracking algorithm needs some time to adapt after sharp changes in frequency, and, during the adaptation, the time-varying band-pass filter is not centered on the underlying oscillatory component.

## Discussion

Advances in analysis methods have revealed the importance of neuronal oscillations in brain activity and (dys)function. Recent studies have highlighted that the top-down control of perception and brain responses is supported to a large extent by oscillatory activity [45]. Consequently, these oscillatory components are now considered as possibly highly efficient information-rich signals in the field of neuroscience. Furthermore, the coupling mechanisms occurring across frequency bands have been the focus of several recent studies [10], [46]–[48]. Collectively, these findings prompted us to develop an adaptive frequency tracking scheme, the SFT, for analyzing EEG data in more detail. Specifically, this algorithm was designed to maximize the oscillatory behavior at the output which is very important for extracting proper phase information, which, in turn, can be used to measure cross-frequency couplings.

The advantages of the SFT for measuring cross-frequency couplings were evaluated with synthetic signals and real EEG data recorded during an IC experiment. First, the synthetic signals in conjunction with Monte Carlo simulations highlighted two desirable features of the proposed algorithm. In the first case, it was shown to be resilient to broad-band noise as the PLV decrease remained limited in high noise levels (Figure 5). In the second case, synthetic signals imitating real EEG recordings were generated in order to check that the SFT could cope well with interfering oscillatory components (Figure 6). Therefore, these numerical simulations illustrated two advantages of the adaptive scheme (resilience to broad-band noise and oscillatory interference) compared to classical filter-bank approaches. These advantages were confirmed when the SFT was applied to real EEG signals for extracting the temporal evolution of differences between the IC and NC conditions in terms of phase-amplitude and phase-phase couplings. The number of successive significant windows was larger with tracking than without for almost all combinations of bands. The advantages of the proposed algorithm were particularly apparent for phase-amplitude couplings. Furthermore, although two significant differences of phase-phase couplings were only detected without the SFT, it led to the detection of five such differences that remained unnoticed with traditional band-pass filtering. And the lengths of the significant intervals were longer with adaptive frequency tracking in most cases. Thus, adaptive frequency tracking could improve the measurements of cross-frequency couplings through precise extraction of neuronal oscillations. Moreover, as the SFT also provides an estimate of the instantaneous frequency of the extracted component, significant changes in frequency could be observed for a few of the bands under study, both when comparing the real and surrogate datasets and the two conditions.

When considering more closely the outcomes of the comparisons between datasets and conditions (Figures 9 and 12), a complex pattern of results was highlighted by the proposed adaptive algorithm. Nonetheless, a few important observations can be pointed out. First, the dataset or condition yielding the highest coupling strength depended on the combination of bands. And, for phase-phase couplings, it also depended on the coupling coefficients, or more specifically on the coefficient ratio. Second, when comparing real and surrogate datasets, the significant differences were in most cases due to changes in coupling strength, either decreases or increases, for the real signals while it remained more or less constant for the surrogate ones. Since the surrogate data were generated so as to be stationary, it was expected. A similar phenomenon was observed when comparing IC and NC conditions. However, usually both conditions elicited a change in coupling strength in the same direction. Nevertheless, this change was typically more pronounced for IC. This seems to indicate that the processing of such contours requires more changes in terms of cross-frequency couplings, but clearly more investigations are needed to confirm this observation. Regarding the outcomes of the comparisons between the two conditions, it is important to mention that the increase of the instantaneous frequency of the 4–8 Hz component observed during IC processing (Figure 8) was too weak to account for the inversion of the differences in phase-phase couplings depending on the coefficient ratio. To summarize, our data provide evidence for condition-wise differences in phase-amplitude and phase-phase couplings. A phase-resetting mechanism [49] might be the cause for these differences. However, the directionality of these differences depended on the considered frequency bands as well as on the selected coupling coefficients, whereas the ERP is generally of higher amplitude for IC than for NC [22]–[24], [26]. Thus, with this dataset, there is no straightforward link between changes in phase locking to a purely phase-resetting model. Consequently, we consider mixed model [50] to be more likely. Clearly, more investigations are required to perfectly understand the role of cross-frequency couplings. Nonetheless, the coupling phenomena reported in this article may link the responses to visual stimuli observed in the lower frequencies [22]–[25], [51], [52] to the ones observed in the higher frequencies [53], alongside the results of numerous studies about cross-frequency couplings [10], [27], [54], [55]. For instance, a recent study [56] investigated with a sustained-attention task the link between cross-frequency couplings and perceptual outcome. In particular, one of the main findings of this study reveals that the relationship between the phase of higher-frequency oscillation and visual-target detection can be almost completely dependent on the phase of delta and theta components.

Some limitations concerning this study and the proposed algorithm are worth discussing. First, the adaptation process in the SFT is not instantaneous, and consequently the estimated frequency suffers some delay. Thus the time-varying band-pass filter used for extraction needs some time to center on the tracked periodic component. This delay not only depends on the SFT parameters, but also on the dynamics of the signal of interest. Indeed, the adaptation is slower in highly non-stationary environments. The delay introduced by the algorithm is clearly visible when comparing the significant intervals for cross-frequency couplings measured with and without adaptive frequency tracking (Figures 12 and 15). A solution to this problem would be to compensate for the delay introduced by the SFT. However, there is no simple technique for this purpose as the delay not only depend on the SFT parameters but also on the input signal dynamics. Therefore, until a reliable approach for delay compensation is developed, cross-frequency couplings could be measured with and without adaptive frequency tracking. The couplings obtained without tracking could help to determine the onset of significant differences, while the ones obtained with the SFT could help to determine the extent of such differences as well as to identify undetected differences. This study also focused on only a small cluster of surface electrodes chosen on the basis of the results of a previous investigation [22], and therefore only local information regarding the cross-frequency couplings were obtained. Ideally, the same analysis procedure should be repeated for all available electrodes in future studies. Doing this will help to explicitly rule out any biases caused by our a priori selection of electrodes. Furthermore, adaptive frequency tracking could also be applied to intracranial EEG signals (which might be difficult to obtain) or to signals computed through inverse solution (e.g. [57]). In particular, the second type of signals can also be used to measure couplings not only across frequency bands but also across different brain areas (e.g. [58]–[60]). However, as the number of signals to analyze increases the processing time may become prohibitive. The computation load comes mainly from the statistical analysis based on permutation tests as the other operations such as filtering and tracking are fairly time-efficient. The coupling analysis performed in this study also raises another important question concerning the direction of the cross-frequency interactions: are the low-frequency oscillations controlling the high-frequency ones or is it the inverse [61]? This issue can be investigated with causality measures, however they still have some drawbacks that may render them inefficient in this case.

## References

[1] Engel AK, Fries P, Singer W (2001) Dynamic predictions: oscillations and synchrony in top-down processing. Nat Rev Neurosci 2: 704–716. 11584308 10.1038/35094565

[2] Varela F, Lachaux JP, Rodriguez E, Martinerie J (2001) The brainweb: phase synchrnization and large-scale integration. Nat Rev Neurosci 2: 229–239. 11283746 10.1038/35067550

[3] Fell J, Axmacher N (2011) The role of phase synchronization in memory processes. Nat Rev Neurosci 12: 105–118. 21248789 10.1038/nrn2979

[4] Fries P (2005) A mechanism for cognitive dynamics: neuronal communication through neuronal coherence. Trends Cogn Sci 9: 474–480. 16150631 10.1016/j.tics.2005.08.011

[5] Axmacher N, Mormann F, Fernández G, Elger CE, Fell J (2006) Memory formation by neuronal synchronization. Brain Res Rev 52: 170–182. 16545463 10.1016/j.brainresrev.2006.01.007

[6] Singer W, Gray CM (1995) Visual feature integration and the temporal correlation hypothesis. Annu Rev Neurosci 18: 555–586. 7605074 10.1146/annurev.ne.18.030195.003011

[7] Engel AK, Singer W (2001) Temporal binding and the neural correlates of sensory awareness. Trends Cogn Sci 5: 16–25. 11164732 10.1016/s1364-6613(00)01568-0

[8] Jensen O, Colgin LL (2007) Cross-frequency coupling between neuronal oscillations. Trends Cogn Sci 11: 267–269. 17548233 10.1016/j.tics.2007.05.003

[9] von Stein A, Sarnthein J (2000) Different frequencies for different scales of cortical integration: from local gamma to long range alpha/theta synchronization. Int J Psychophysiol 38: 301–313. 11102669 10.1016/s0167-8760(00)00172-0

[10] Canolty RT, Edwards E, Dalal SS, Soltani M, Nagarajan SS, et al (2006) High gamma power is phase-locked to theta oscillations in human neocortex. Science 313: 1626–1628. 16973878 10.1126/science.1128115 PMC2628289

[11] Canolty RT, Ganguly K, Kennerley SW, Cadieu CF, Koepsell K, et al (2010) Oscillatory phase coupling coordinates anatomically dispersed functional cell assemblies. P Natl Acad Sci USA 107: 17356–17361. 10.1073/pnas.1008306107 PMC2951408 20855620

[12] Lakatos P, Chen CM, O'Connell MN, Mills A, Schroeder CE (2007) Neuronal oscillations and multisensory interaction in primary auditory cortex. Neuron 53: 279–292. 17224408 10.1016/j.neuron.2006.12.011 PMC3717319

[13] Lakatos P, Karmos G, Mehta AD, Ulbert I, Schroeder CE (2008) Entrainment of neuronal oscilla- tions as a mechanism of attentional selection. Science 320: 110–113. 18388295 10.1126/science.1154735

[14] Demiralp T, Bayraktaroglu Z, Lenz D, Junge S, Busch NA, et al (2007) Gamma amplitudes are coupled to theta phase in human EEG during visual perception. Int J Psychophysiol 64: 24–30. 16956685 10.1016/j.ijpsycho.2006.07.005

[15] Sauseng P, Klimesch W, Heise KF, Gruber WR, Holz E, et al (2009) Brain oscillatory substrates of visual short-term memory capacity. Curr Biol 19: 1846–1852. 19913428 10.1016/j.cub.2009.08.062

[16] Foster BL, Parvizi J (2012) Resting oscillations and cross-frequency coupling in the human pos- teromedial cortex. Neuroimage 60: 384–391. 22227048 10.1016/j.neuroimage.2011.12.019 PMC3596417

[17] Buzsáki G, Wang XJ (2012) Mechanisms of gamma oscillations. Annu Rev Neurosci 35: 203–225. 22443509 10.1146/annurev-neuro-062111-150444 PMC4049541

[18] Kanizsa G (1976) Subjective contours. Sci Am 234: 48–52. 1257734 10.1038/scientificamerican0476-48

[19] Tallon-Baudry C, Bertrand O, Delpuech C, Pernier J (1996) Stimulus specificity of phase-locked and non-phase-locked 40 Hz visual responses in human. J Neurosci 16: 4240–4249. 8753885 10.1523/JNEUROSCI.16-13-04240.1996 PMC6579008

[20] Herrmann CS, Mecklinger A, Pfeifer E (1999) Gamma responses and ERPs in a visual classification task. Clin Neurophysiol 110: 636–642. 10378732 10.1016/s1388-2457(99)00002-4

[21] Csibra G, Davis G, Spratling MW, Johnson MH (2000) Gamma oscillations and object processing in the infant brain. Science 290: 1582–1585. 11090357 10.1126/science.290.5496.1582

[22] Murray MM, Wylie GR, Higgins BA, Javitt DC, Schroeder CE, et al (2002) The spatiotemporal dynamics of illusory contour processing: combined high-density electrical mapping, source analysis, and functional magnetic resonance imaging. J Neurosci 22: 5055–5073. 12077201 10.1523/JNEUROSCI.22-12-05055.2002 PMC6757726

[23] Murray MM, Foxe DM, Javitt DC, Foxe JJ (2004) Setting boundaries: brain dynamics of modal and amodal illusory shape completion in humans. J Neurosci 24: 6898–6903. 15295024 10.1523/JNEUROSCI.1996-04.2004 PMC6729598

[24] Murray MM, Imber ML, Javitt DC, Foxe JJ (2006) Boundary completion is automatic and disso- ciable from shape discrimination. J Neurosci 26: 12043–12054. 17108178 10.1523/JNEUROSCI.3225-06.2006 PMC6674876

[25] Shpaner M, Murray MM, Foxe JJ (2009) Early processing in the human lateral occipital complex is highly responsive to illusory contours but not to salient regions. Eur J Neurosci 30: 2018–2028. 19895562 10.1111/j.1460-9568.2009.06981.x PMC3224794

[26] Knebel JF, Murray MM (2012) Towards a resolution of conicting models of illusory contour processing in humans. Neuroimage 59: 2808–2817. 21979384 10.1016/j.neuroimage.2011.09.031

[27] Bosman CA, Zamorano F, Aboitiz F (2010) Functional differences of low- and high-frequency oscillatory dynamics during illusory border perception. Brain Res 1319: 92–102. 20064488 10.1016/j.brainres.2010.01.004

[28] Tass P, Rosenblum MG, Weule J, Kurths J, Pikovsky A, et al (1998) Detection of n: m phase locking from noisy data: application to magnetoencephalography. Phys Rev Lett 81: 3291–3294.

[29] Lachaux JP, Rodriguez E, Martinerie J, Varela FJ (1999) Measuring phase synchrony in brain signals. Hum Brain Mapp 8: 194–208. 10619414 10.1002/(SICI)1097-0193(1999)8:4<194::AID-HBM4>3.0.CO;2-C PMC6873296

[30] Schyns PG, Thut G, Gross J (2011) Cracking the code of oscillatory activity. PLoS Biol 9: e1001064. 21610856 10.1371/journal.pbio.1001064 PMC3096604

[31] Gabor D (1946) Theory of communication. J IEE 93: 429–457.

[32] Chavez M, Besserve M, Adam C, Martinerie J (2006) Towards a proper estimation of phase syn- chronization from time series. J Neurosci Meth 154: 149–160. 10.1016/j.jneumeth.2005.12.009 16445988

[33] Celka P (2007) Statistical analysis of the phase-locking value. IEEE Signal Proc Let 14: 577–580.

[34] Uldry L, Duchêne C, Prudat Y, Murray MM, Vesin JM (2009) Adaptive tracking of EEG frequency components. In: Nat-Ali A, editor, Advanced biosignal processing, Springer. pp. 123–144.

[35] Van Zaen J, Uldry L, Duchêne C, Prudat Y, Meuli RA, et al (2010) Adaptive tracking of EEG oscillations. J Neurosci Meth 186: 97–106. 10.1016/j.jneumeth.2009.10.018 19891985

[36] Oldfield RC (1971) The assessment and analysis of handedness: the Edinburgh inventory. Neu- ropsychologia 9: 97–113. 10.1016/0028-3932(71)90067-4 5146491

[37] Brunet D, Murray MM, Michel CM (2011) Spatiotemporal Analysis of Multichannel EEG: CAR- TOOL. Comput Intell Neurosci 2011: 813870. 21253358 10.1155/2011/813870 PMC3022183

[38] Oppenheim AV, Schafer RW (2010) Discrete-time signal processing. Pearson, third edition

[39] Marple SL (1999) Computing the discrete-time analytic signal from the FFT. IEEE T Signal Proces 47: 2600–2603.

[40] Schreiber T, Schmitz A (2000) Surrogate time series. Physica D 142: 346–382.

[41] Borgnat P, Flandrin P, Honeine P, Richard C, Xiao J (2010) Testing stationarity with surrogates: a time-frequency approach. IEEE T Signal Proces 58: 3459–3470.

[42] Billingsley P (1995) Probability and measure. John Wiley & Sons, third edition

[43] Penny WD, Duzel E, Miller KJ, Ojemann JG (2008) Testing for nested oscillation. J Neurosci Meth 174: 50–61. 10.1016/j.jneumeth.2008.06.035 PMC2675174 18674562

[44] Efron B, Tibshirani RJ (1993) An introduction to the bootstrap. Chapman & Hall

[45] Buzsáki G (2006) Rhythms of the brain. Oxford University Press.

[46] de Lange FP, Jensen O, Bauer M, Toni I (2008) Interactions between posterior gamma and frontal alpha/beta oscillations during imagined actions. Front Hum Neurosci 2: 7. 18958208 10.3389/neuro.09.007.2008 PMC2572199

[47] Osipova D, Hermes D, Jensen O (2008) Gamma power is phase-locked to posterior alpha activity. PLoS ONE 3: e3990. 19098986 10.1371/journal.pone.0003990 PMC2602598

[48] Schroeder CE, Lakatos P (2009) Low-frequency neuronal oscillations as instruments of sensory selection. Trends Neurosci 32: 9–18. 19012975 10.1016/j.tins.2008.09.012 PMC2990947

[49] Makeig S, Debener S, Onton J, Delorme A (2004) Mining event-related brain dynamics. Trends Cogn Sci 8: 204–210. 15120678 10.1016/j.tics.2004.03.008

[50] Shah AS, Bressler SL, Knuth KH, Ding M, Mehta AD, et al (2004) Neural dynamics and the fundamental mechanisms of event-related brain potentials. Cereb Cortex 14: 476–483. 15054063 10.1093/cercor/bhh009

[51] Foxe JJ, Murray MM, Javitt DC (2005) Filling-in in schizophrenia: a high-density electrical map- ping and source-analysis investigation of illusory contour processing. Cereb Cortex 15: 1914–1927. 15772373 10.1093/cercor/bhi069

[52] Knebel JF, Javitt DC, Murray MM (2011) Impaired early visual response modulations to spatial information in chronic schizophrenia. Psychiat Res-Neuroim 193: 168–176. 10.1016/j.pscychresns.2011.02.006 PMC3156880 21764264

[53] Tallon-Baudry C, Bertrand O (1999) Oscillatory gamma activity in humans and its role in object representation. Trends Cogn Sci 3: 151–162. 10322469 10.1016/s1364-6613(99)01299-1

[54] Buzsáki G, Draguhn A (2004) Neuronal oscillations in cortical networks. Science 304: 1926–1929. 15218136 10.1126/science.1099745

[55] Canolty RT, Knight RT (2010) The functional role of cross-frequency coupling. Trends Cogn Sci 14: 506–515. 20932795 10.1016/j.tics.2010.09.001 PMC3359652

[56] Fiebelkorn IC, Snyder AC, Mercier MR, Butler JS, Molholm S, et al (2013) Cortical cross-frequency coupling predicts perceptual outcomes. Neuroimage 69: 126–137. 23186917 10.1016/j.neuroimage.2012.11.021 PMC3872821

[57] Michel CM, Murray MM (2012) Towards the utilization of EEG as a brain imaging tool. Neuroim- age 61: 371–385. 10.1016/j.neuroimage.2011.12.039 22227136

[58] Astolff L, Toppi J, De Vico Fallani F, Vecchiato G, Salinari S, et al (2010) Neuroelectrical hyper- scanning measures simultaneous brain activity in humans. Brain Topogr 23: 243–256. 20480221 10.1007/s10548-010-0147-9

[59] De Vico Fallani F, Rodrigues F, da Fontoura Costa L, Astolfi L, Cincotti F, et al (2011) Multiple pathways analysis of brain functional networks from EEG signals: an application to real data. Brain Topogr 23: 344–354. 20614232 10.1007/s10548-010-0152-z

[60] Zalesky A, Cocchi L, Fornito A, Murray MM, Bullmore E (2012) Connectivity differences in brain networks. Neuroimage 60: 1055–1062. 22273567 10.1016/j.neuroimage.2012.01.068

[61] Schroeder CE, Lakatos P (2009) The gamma oscillation: master or slave? Brain Topogr 22: 24–26. 19205863 10.1007/s10548-009-0080-y PMC2989849

